# The Canadian Chronic Disease Surveillance System: The Benefits and Challenges of a Distributed Model for National Disease Surveillance

**DOI:** 10.23889/ijpds.v1i1.356

**Published:** 2017-04-19

**Authors:** Lisa Lix, Kim Reimer

**Affiliations:** 1 University of Manitoba; 2 British Columbia Ministry of Health

## Objectives

The Public Health Agency of Canada (PHAC) established the Canadian Chronic Disease Surveillance System (CCDSS) in 2009 to facilitate national estimates of chronic disease prevalence, incidence, and health outcomes. The CCDSS uses population-based linked health administrative databases from all provinces/territories (P/Ts) and a distributed analytic protocol to produce standardized disease estimates. Our purpose is to describe the process, benefits, and challenges of implementing a distributed model for disease surveillance across thirteen jurisdictions with unique healthcare databases.

## Approach

The CCDSS is founded on deterministic linkage of three administrative health databases in each Canadian P/T: health insurance registration files, physician billing claims, and hospital discharge abstracts. Disease case definitions are developed by expert Working Groups after literature reviews are completed and validation studies are undertaken. Feasibility studies are initiated in selected P/Ts to identify challenges when implementing the disease case definitions. Analytic code developed by PHAC is then distributed to all P/Ts. Data quality surveys are routinely conducted to identify database characteristics that may bias disease estimates over time or across P/Ts or affect implementation of the analytic code. The summary data produced in each P/T are approved by Scientific Committee and Technical Committee members and then submitted to PHAC for further analysis and reporting.

## Results

National surveillance or feasibility studies are currently ongoing for diabetes, hypertension, selected mental illnesses, chronic respiratory diseases, heart disease, neurological conditions, musculoskeletal conditions, and stroke. The advantages of the distributed analytic protocol are: (a) changes in methodology can be easily made, and (b) technical expertise to implement the methodology is not required in each P/T. Challenges in the use of the distributed analytic protocol are: (a) heterogeneity in healthcare databases across P/Ts and over time, (b) the requirement that each P/T use the minimum set of data elements common to all jurisdictions when producing disease estimates, and (c) balancing disclosure guidelines to ensure data confidentiality with comprehensive reporting. Additional challenges, which include incomplete data capture for some databases and poor measurement validity of disease diagnosis codes for some chronic conditions, must be continually addressed to ensure the scientific rigor of the CCDSS methodology.

## Conclusions

The CCDSS distributed analytic protocol offers one model for national chronic disease surveillance that has been successfully implemented and sustained by PHAC and its P/T partners. Many lessons have been learned about national chronic disease surveillance involving jurisdictions that are heterogeneous with respect to healthcare databases, expertise, and population characteristics.

